# Hydrogels for Bioprinting: A Systematic Review of Hydrogels Synthesis, Bioprinting Parameters, and Bioprinted Structures Behavior

**DOI:** 10.3389/fbioe.2020.00776

**Published:** 2020-08-06

**Authors:** Enrique Mancha Sánchez, J. Carlos Gómez-Blanco, Esther López Nieto, Javier G. Casado, Antonio Macías-García, María A. Díaz Díez, Juan Pablo Carrasco-Amador, Diego Torrejón Martín, Francisco Miguel Sánchez-Margallo, J. Blas Pagador

**Affiliations:** ^1^Bioengineering and Health Technologies Unit, Minimally Invasive Surgery Centre Jesús Usón, Cáceres, Spain; ^2^Stem Cells Unit, Minimally Invasive Surgery Centre Jesús Usón, Cáceres, Spain; ^3^School of Industrial Engineering, University of Extremadura, Badajoz, Spain; ^4^Scientific Direction, Minimally Invasive Surgery Centre Jesús Usón, Cáceres, Spain

**Keywords:** bioprinting, hydrogel, systematic review, biomaterial, bioink

## Abstract

Nowadays, bioprinting is rapidly evolving and hydrogels are a key component for its success. In this sense, synthesis of hydrogels, as well as bioprinting process, and cross-linking of bioinks represent different challenges for the scientific community. A set of unified criteria and a common framework are missing, so multidisciplinary research teams might not efficiently share the advances and limitations of bioprinting. Although multiple combinations of materials and proportions have been used for several applications, it is still unclear the relationship between good printability of hydrogels and better medical/clinical behavior of bioprinted structures. For this reason, a PRISMA methodology was conducted in this review. Thus, 1,774 papers were retrieved from PUBMED, WOS, and SCOPUS databases. After selection, 118 papers were analyzed to extract information about materials, hydrogel synthesis, bioprinting process, and tests performed on bioprinted structures. The aim of this systematic review is to analyze materials used and their influence on the bioprinting parameters that ultimately generate tridimensional structures. Furthermore, a comparison of mechanical and cellular behavior of those bioprinted structures is presented. Finally, some conclusions and recommendations are exposed to improve reproducibility and facilitate a fair comparison of results.

## Introduction

Biofabrication can be defined as a multidisciplinary research field with a combination of principles, protocols, and fabrication techniques from engineering, electronics, material science, cell biology, and biomedicine (Silva, 2018). Bioprinting is a biofabrication technique that can control deposition of cells, extracellular matrix components, and biochemical factors layer by layer to create defined structures with several kinds of materials, bioactive molecules, and cells (Moroni et al., 2018; Eswaramoorthy et al., 2019). In this sense, bioprinting allows the generation of complex structures mimicking biological cues, which increases the possibilities of tissue creation by supporting and improving other traditional techniques of tissue engineering (Moldovan, 2019). Besides, all bioprinting techniques are also constantly and rapidly evolving thanks to the advances in technical processes and bioink (hydrogel with cells in culture medium) properties (Silva, 2018).

Synthesis, bioprinting, and cross-linking of bioinks have a great impact on the generation of biological structures and especially on its mechanical and cellular behavior. Therefore, bioink is one of the most critical components of 3D bioprinting and it is intimately related to the bioprinting technique and the selected cells (Kyle et al., 2017).

Although there are many bioprinting techniques, such as laser, inkjet, droplet, stereolithography, and electrospinning (Leberfinger et al., 2019), we have focused this review on extrusion-based bioprinting. This technique is widely used by researchers, mainly due to its low cost and versatility that allow mechanical modifications and a wide range of materials, but above all high cell densities (Kyle et al., 2017; Jovic et al., 2019; Leberfinger et al., 2019). It uses the widest range of biomaterials including hydrogels, biocompatible copolymers, and cell spheroids that have many different printability properties, such as viscosity, density, or shear thinning properties, among others.

Each bioprinting procedure needs a specific set of rheological and mechanical properties of the bioink to achieve a successful bioprinted structure. On the one hand, extrusion-based process must control the properties referred to shear thinning, like viscosity and shear stress, to mitigate cell damage. On the other hand, inkjet (droplet-based) process must control surface tension and viscosity of bioinks to get a proper droplet ejection (Leberfinger et al., 2019).

In this review, natural and synthetic materials used to produce hydrogels with different features and behaviors have been analyzed. According to the bioprinting process, those important parameters involved in the bioprinter setting have been exposed (He et al., 2016; Sodupe-Ortega et al., 2018). Finally, tests for validation of bioprinted structures have been included and grouped into two blocks: cell and mechanical properties.

**Figure 1:**
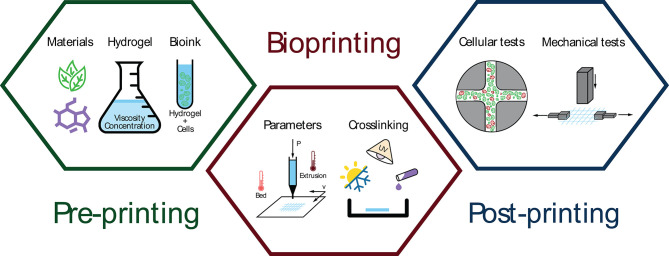
Schematic representation of three bioprinting stages: pre-printing (material selection, hydrogel synthesis, and bioink generation), extrusion-based bioprinting (parameters and cross-linking methods), and post-printing analysis (cellular and mechanical tests).

Hence, the main goal of this systematic review is to analyze the impact of pre-printing stage (materials selection, hydrogel synthesis, and bioink generation) and extrusion-based bioprinting process (both bioprinting parameters and cross-linking methods) on post-printing results of the bioprinted structures (cell tests and mechanical properties) (Figure 1).

## Materials and Methods

### Identification of Peer-Reviewed Manuscripts for Analysis Literature

A systematic review was conducted in accordance with PRISMA (P) guidelines. This review included original peer-reviewed studies based on the following criteria: (1) publication in an English-language journal; (2) papers related to extrusion-based bioprinting; (3) only original peer-reviewed papers were included, so editorials, proceedings, communications, letters, and reviews were excluded; (4) all papers based on complex tissue generation, organ generation or drug testing were excluded.

The search engines used to identify studies were PUBMED, WOS, and SCOPUS. The following search items were used for the literature search: (Bioprinting AND Hydrogel). Search was performed on April 15th, 2019. No restriction was set on publication date. No meta-analyses were performed due to the heterogeneity of studies.

### Data Extraction and Analysis

Before reviewing papers, several categories for data extraction were defined. A form was created and divided into different categories: (1) ***general data***compiles authors, title, publication year, and journal; (2) ***material***categorizes different materials by their importance and/or functionalities, including concentration and viscosity; (3) ***printing settings***gathers cartridge temperature, bed temperature, printing pressure, and printing speed; (4) ***cross-linking methods***summarizes cross-linking process depending on its type and characteristics, and finally (5) ***validation tests***registers types of cellular viability and mechanical tests. Specifically, *main materials* were classified as synthetic or natural, material name, cell-laden or post-printing cell addition, and according to its type of cross-linking (thermal, chemical, or physical). Furthermore, *structural material* was subdivided on material name and cross-linking type. Finally, *sacrificial material* was defined by material name, cross-linking type, and removal process. Papers were individually assigned to eight independent reviewers to be read in detail to extract available data.

## Results and Discussion

### Overall Findings

**Figure 2:**
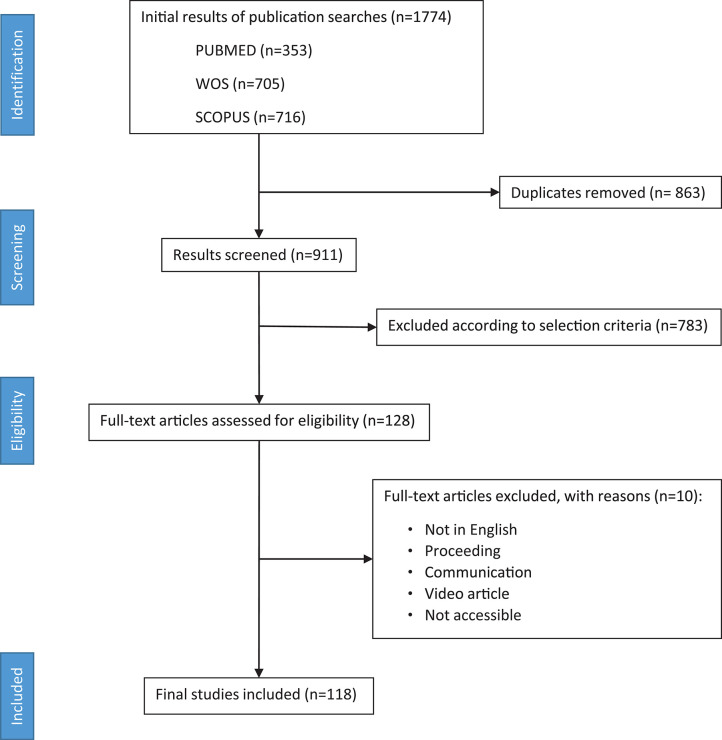
PRISMA flow diagram depicting literature search, exclusion process, eligibility criteria, and final included papers. One hundred and eighteen papers were included without publication date restriction (search performed on April 15th, 2019).

In all, 1,774 abstracts were found using the search string (Bioprinting AND Hydrogel) in three databases (PUBMED, WOS, and SCOPUS). Nine hundred and eleven papers were screened after removing duplicated, 783 of them excluded according to selection criteria, and 128 revised in full text. From those, 118 were finally considered for the review analysis (Figure 2).

**Figure 3:**
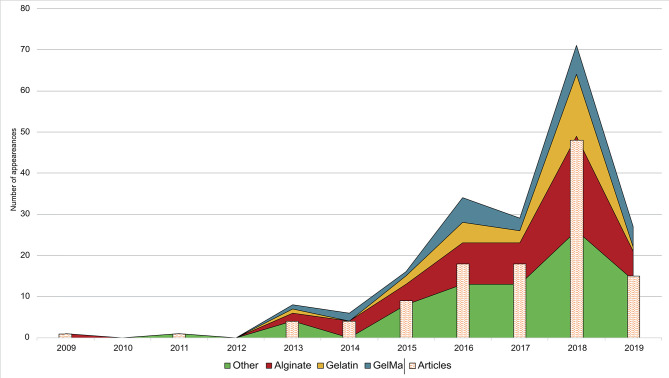
Research trend in hydrogels for bioprinting using a bar chart (*papers per year*) combined with a stacked area chart (*total uses of a material per year*). The three most used materials (alginate, gelatin, and GelMA) are shown individually while the rest of materials are grouped in the category “other.” It is important to note that some materials are used in more than one paper, so some stacked areas overtake its corresponding bar chart.

Figure 3 shows an upward trend in the number of published papers during the last decade. There were no papers prior to 2009, only one published in this year, and an increase from 2015 onwards. However, 2017 meant a year of stagnation that could be due to an increase of research studies focused on the creation of more complex tissues, organoids, drug testing, and lab-on-a chip (Ma J. et al., 2018; Ma X. et al., 2018; Reid et al., 2019), subjects that are out of the scope of this review. In 2018, the research community came back to the creation of new materials and structures. These studies could provide better results in terms of cellular viability, histo-differentiation of complex tissues and the formation of complex structures. On the other hand, a crucial point could rely on a higher accessibility to low-cost or home-made bioprinters (Ozbolat et al., 2017).

Additionally, Figure 3 shows annual papers regarding the three most used materials as main component (alginate, gelatin, and GelMA) whereas Furthermore, the rest of materials was grouped in the category “other.” Material trend is similar to year trend, with a few differences. In 2014 and 2017, total number of used materials is lower in comparison to previous years, which means that fewer papers used more than one material. On the other hand, papers published in the first trimester of 2019 showed a rising forecast for this year. It is difficult to make an approach to what kind of papers will be published in upcoming years, but everything indicates that new synthetic materials and mixtures of other complex materials could grow up (Ashammakhi et al., 2019).

### Journals Analysis

In this section, a classification of journals has been made to analyze what type of publications deal with our topics. Four main categories and other four subcategories from JCR or SJR were used to group journals. The main categories are: (1) ***material***, journals of chemical/material-centered issues; (2) ***cellular***, journals focused on the cellular/histological/biochemical topic; (3) ***engineering***, journals focused on the technical and/or mechanical issues, and (4) ***multidisciplinary***, journals that allow multiple topics. Additionally, the four combined subcategories are: (1) **engineering/material**, (2) **cellular/material**, (3) **engineering/cellular**, and (4) **engineering/material/cellular**.

**Figure 4:**
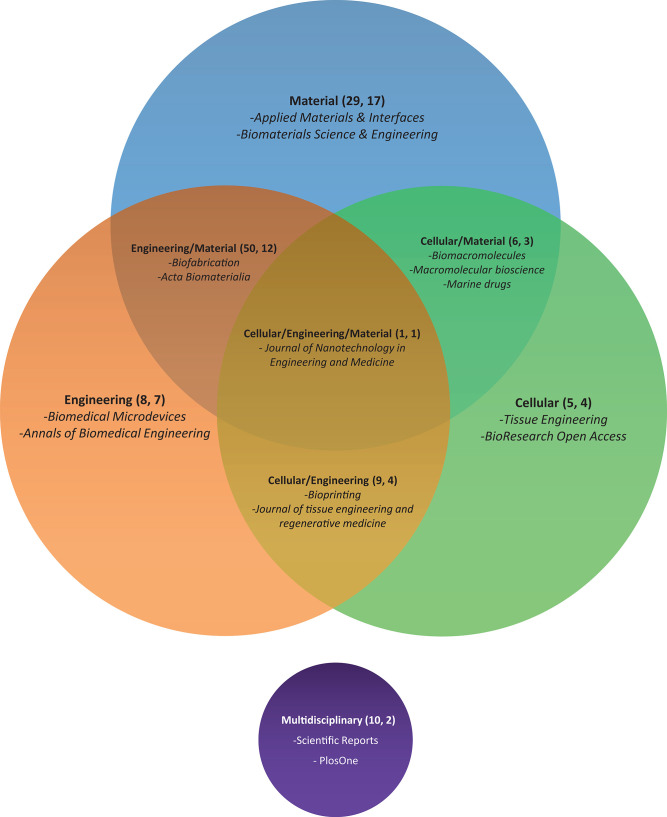
Venn diagram of journals' categories selected in this review according to JCR/SJR categories. Diagram information is organized as follows: Topic of the journal (number of papers, number of journals) and some of the most represented journals are listed. We noted that intersection areas are exclusive, and sizes are not proportional.

Figure 4 shows distribution of all 50 journals. **Material** contains the highest number of journals with 17 (29 papers). “Applied Materials & Interfaces” and “Biomaterials Science & Engineering” are the two most common journals in this category with 6 and 4 papers, respectively. All combinations of the **material** category reach 33 journals (86 papers). The remaining categories contain fewer journals. **Cellular** and **engineering** categories includ only four and seven journals (5 and 8 papers), respectively. “Scientific Reports” and “Plos One” are two journals associated to **multidisciplinary** category with 8 and 3 papers, respectively. “Journal of nanotechnology in Engineering and Medicine” is the only journal associated to the **engineering/material/cellular** category with one paper. Finally, the **material/engineering** subcategory is by far the most common with 12 journals (50 papers). In this subcategory, most papers are published by “Biofabrication” (23 papers) and “Acta Biomateralia” (8 papers).

### Hydrogel Generation (Pre-printing)

#### Natural vs. Synthetic

Natural and synthetic polymers can be considered as a broad cataloging of materials to synthesize hydrogels. In this sense, natural polymers are defined as bio-derived materials present in nature that can be extracted using physical or chemical methods (e.g., gelatin, alginate, or chitosan). On the other hand, those human-made polymers are named synthetic and are usually classified into plastics, elastomers, and synthetic fibers (Ouellette and Rawn, 2015).

**Figure 5:**
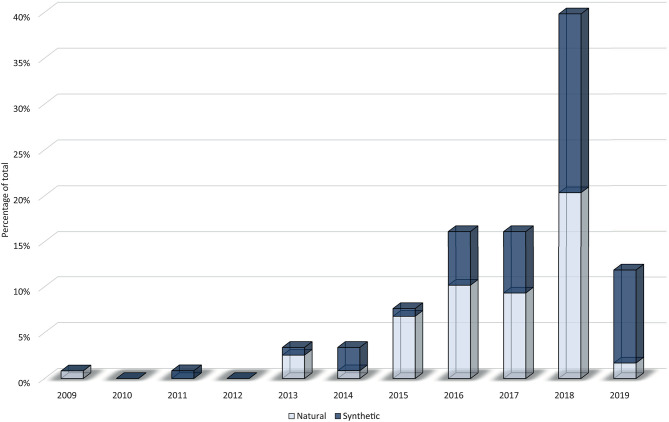
Stacked columns graph that shows evolution per year of natural (white) and synthetic (blue) materials on the percentage of total.

In general, authors use natural materials more than synthetic (Figure 5) due to their better biological properties (Silva, 2018) at the expense of the best mechanical properties of synthetic materials (Abelardo, 2018). A chronological classification of papers show few studies between 2009 and 2014 (10 out of 118) followed by a huge increment in the use of natural materials in 2015 (89% of all papers in this year). After that, natural materials clearly have a downward trend in favor of synthetic materials that reached 36, 42, 48, and 85% throughout 2016, 2017, 2018, and 2019, respectively. Maybe this trend is due to high biocompatibility and affordable price of natural materials during the first years of bioprinting. However, rheological properties of natural materials are not the best for printability, and mechanical properties of the bioprinted structures are only appropriate for some applications. For this reason, once these natural materials reached their technical and biological limitations, the use of synthetic materials began to rise in order to solve these former problems.

#### Materials

The selection of materials is one of the most important decisions for the hydrogel generation. They have a great impact on biocompatibility and cellular viability as well as the mechanical behavior of the bioprinted structures, what is mandatory for a good bioprinting result. In this sense, all 118 papers used 34 different materials, although some chemical modifications were performed in some of them (e.g., alginate with norbornene) that are not considered as different material in this review. Specifically, the most common materials was alginate appearing in 58 papers followed by gelatin (26), GelMA (25), hyaluronic acid (16), and polyethylene glycol (PEG) with its chemical modifications (16).

Although complex tissues and organs generation are out of the scope of this review, we consider interesting to include some information about those papers that define its biological purpose (61 out of 118). Most of them have a low frequency or a generic soft tissue use, but cartilage (22 papers) and vascular (9) usually use alginate (12 and 6 papers) and GelMA (9 and 2 papers), respectively.

**Figure 6:**
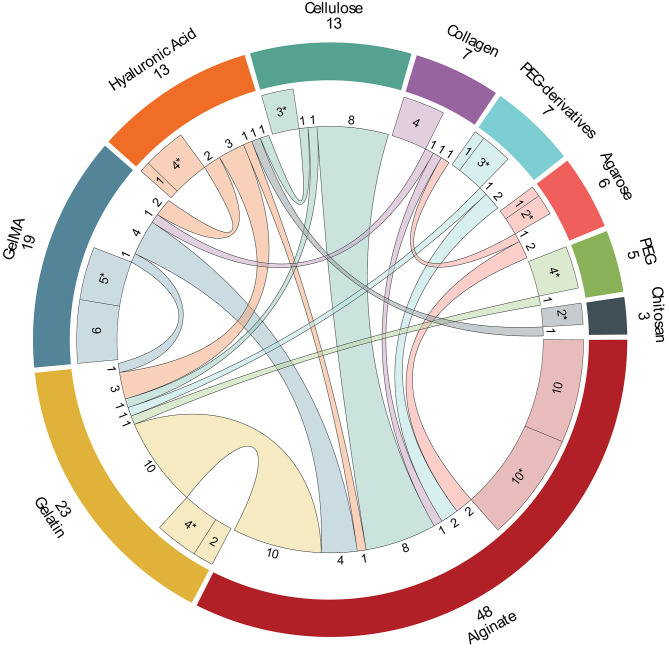
Combination of the 10 most used materials in hydrogels among them. The materials are shown in the external ring (total of papers and name). The middle ring segments represent one-material hydrogels and hydrogels, marked with (*), that are a combination of this material with non-selected materials (one-material papers mixed with non-selected materials papers). Inner lines represent hydrogels with two of the selected materials, but in some case other non-selected materials can be included (number of papers next to each inner line).

In this review, the 10 most used materials were selected for a detailed analysis. Figure 6 shows the combination of these 10 materials in different hydrogels (103 papers). However, in order to make clear this figure, those papers that use hydrogels with more than two of these selected materials (12) and papers that use materials different of these ten (3) were excluded from this figure and analyzed later in this section.

*Alginate* is the most used material in bioprinting appearing in a total of 58 papers. It is used with the other selected materials in pairs in 28 papers, with more than two selected materials in 10 papers, alone in 10 papers and with other chemical modifications in other 10 papers. Some of these interesting chemical modifications that improve its characteristics are: oxidized alginate (ox-alg) (Hafeez et al., 2018) which gives alginate a faster degradation and more reactive groups (Boontheekul et al., 2005), methacrylated alginate (MeAlg/AlgMA) which allows photo-polymerization thanks to its methacryloyl groups (García-Lizarribar et al., 2018; Ji et al., 2019), both oxidized and methacrylated together (ox-MeAlg) (Jeon et al., 2019), and alginate with norbornene (alg-norb) (Ooi et al., 2018) which provides alginate an ultrafast light-triggered cross-linking. Firstly, gelatin is the hydrogel which appears more times with alginate in just two materials combinations (Chung et al., 2013; He et al., 2016; Wang et al., 2016; Ding et al., 2017, 2018; Giuseppe et al., 2017; Aljohani et al., 2018; Berg et al., 2018; Gao et al., 2018; Li et al., 2018c). It is important to note that alginate/gelatin combination allows hydrogel to have good rheological properties (alginate) with proper thermoresponsive behavior (gelatin). Secondly, different types of cellulose have been used in combination with alginate: cellulose nanocrystals (CNC) (Habib et al., 2018; Wu Y. et al., 2018), methylcellulose (Li et al., 2017; Schütz et al., 2017; Ahlfeld et al., 2018; Gonzalez-Fernandez et al., 2019), and nanofibrillated cellulose (NFC) (Markstedt et al., 2015; Apelgren et al., 2017). Gelatin and cellulose are followed by GelMA (Liu et al., 2018; Zhang X. et al., 2018; Kosik-Kozioł et al., 2019; Krishnamoorthy et al., 2019), PEG-derived (Maiullari et al., 2018; Yu et al., 2018), agarose (Blaeser et al., 2013; López-Marcial et al., 2018), hyaluronic acid (Ji et al., 2019), and collagen (Campbell et al., 2015) to produce hydrogels of two materials. The rest of studies uses three and four-materials hydrogels with 7 and 3 papers, respectively. It is particularly interesting that 5 out of these 10 papers utilized alginate and GelMA with other different components: hyaluronic acid methacrylated (Costantini et al., 2016), PEG (Daly et al., 2016b), PEGDA (Kang et al., 2017), PEDGA/cellulose (García-Lizarribar et al., 2018), and PEGMA/agarose (Daly et al., 2016a). In the same way, four hydrogels are composed by alginate and collagen with different components: gelatin (Bandyopadhyay et al., 2018), chitosan (Aydogdu et al., 2019), agarose (Yang et al., 2017), and gelatin/chitosan (Akkineni et al., 2016). Finally, other hydrogel is composed by alginate, hyaluronic acid, and cellulose (Nguyen et al., 2017). Despite the lack of good rheological properties of natural materials, alginate is one of the best natural polymer in rheology. Additionally, good biocompatibility and its facility to form reticular structures using Ca^2+^ ions have popularized its use in bioprinting (Lee and Mooney, 2012).

*Gelatin* is the second most used material with 26 papers. It is a component of hydrogels with other selected materials (17 papers) and with other combinations (7) mainly due to its poor rheological properties. Maybe for this reason, it is used alone in just two papers (Choi et al., 2018; Tijore et al., 2018b). In other studies, several modifications have been performed to enhance its properties. So, furfuryl-gelatin (f-gelatin) allows cross-linking with visible light (AnilKumar et al., 2019), but other studies combined with hyaluronic acid (Zhang K. et al., 2016; Shin et al., 2018; AnilKumar et al., 2019), GelMA (McBeth et al., 2017), cellulose (Xu X. et al., 2018), PEGDA (Aied et al., 2018), and PEG (Irvine et al., 2015). Additionally, it is also used in three-materials (Bandyopadhyay et al., 2018; Haring et al., 2019) and four-materials (Akkineni et al., 2016) hydrogels. Specifically, two important features of gelatin can be remarked: cellular adhesion, mainly due to presence of RGD (arginine-glycine-aspartate) sequences, and thermoresponsive behavior that sustains the use of gelatin as supporting material.

*GelMA* is the third material and the first synthetic one, compose by gelatin with methacrylated groups (Billiet et al., 2014). In general, GelMA has excellent rheological properties that improve printability, shape fidelity, and stability of the hydrogel due to UV photopolymerization of its methacrylated groups (Pepelanova et al., 2018). For this reason, modifications of this material are unusual and only Haring et al. (2019) modifieds GelMA with a dopamine molecule. Hence, it appears in 25 papers, it is combined with other selected material in 8 papers, used alone in othersix (Bertassoni et al., 2014; Billiet et al., 2014; Ersumo et al., 2016; Gu et al., 2018; Pepelanova et al., 2018; Zhou et al., 2019), with more than two of the selected materials, and used with other different materials in 5 papers. Alginate is the most common combination of GelMA appearing in 4, 3, and 2 papers for two-, three-, and four-material hydrogels, respectively (further details in the alginate section). Other common materials used with GelMA are hyaluronic acid (Schuurman et al., 2013; Noh et al., 2019), gelatin (McBeth et al., 2017), and collagen (Du et al., 2015). The rest of papers combines GelMA with pluronic (Levato et al., 2017; Suntornnond et al., 2017), carrageenan (Li et al., 2018b,c), gellan gum (Mouser et al., 2016), and polyisocyanide (PIC) (Celikkin et al., 2018).

*Hyaluronic acid (HA)* and its derived materials are quite used being the fourth material in the list with 16 papers. It is an anionic, non-sulfated glycosaminoglycan that is present in connective and neural tissues as well as it is one of the major components of the skin. For this reason, HA is mostly used in skin tissue engineering. It appears alone in five papers (Mouser et al., 2017; Stichler et al., 2017; Lee et al., 2018; Wang et al., 2018; Kiyotake et al., 2019). Furthermore, there are three studies that use HA combined with gelatin (Zhang K. et al., 2016; Shin et al., 2018; AnilKumar et al., 2019) but other authors use GelMA (Schuurman et al., 2013; Noh et al., 2019), alginate/cellulose (Law et al., 2018), and chitosan (Kim et al., 2019). This material is modified in half of the papers, obtaining: hyaluronic acid methacrylated (HAMA) (Costantini et al., 2016; Mouser et al., 2017), acrylated-HA and tyramine-conjugated HA (Lee et al., 2018), dopamine-conjugated HA (Haring et al., 2019), and thiol functionalized HA (Stichler et al., 2017). Concretely, HAMA provides great tunability for specific uses at different methacrylation degrees (Xia et al., 2017).

*Cellulose* is the following material with 15 papers. Cellulose fibers are obtained from natural resources and are widely used in bioprinting to improve mechanical properties of hydrogels. Depending on the polymerization degree, the length of its polymeric chain, hydrogels with cellulose can have from high tensile strength (long chains) to solubility properties (short chains). It is usually modified replacing some hydroxyl groups with methoxy groups forming methylcellulose. Specifically, cellulose is used alone in three papers (Béduer et al., 2018; Cochis et al., 2018; Contessi Negrini et al., 2018). Additionally, it is combined with alginate (detailed above), gelatin (Xu X. et al., 2018), or hyaluronic acid (Law et al., 2018), being the two remaining papers combinations of alginate/cellulose with hyaluronic acid (Nguyen et al., 2017) and GelMA/PEGDA (García-Lizarribar et al., 2018).

*Collagen* is other popular material in bioprinting appearing in 12 papers that is the main component of the extracellular matrix, e.g., connective tissues as cartilage. In this review, collagen is used alone in four papers (Hartwell et al., 2016; Kim et al., 2016; Ren et al., 2016; Ahn et al., 2017)and appears in combination with alginate (Campbell et al., 2015), GelMA (Du et al., 2015), and agarose (Köpf et al., 2016). The rest of combinations with more material was described in previous sections.

*Polyethylene glycol (PEG)* and its derivatives PEG diacrylated (PEGDA) and PEG methacrylated (PEGMA) are used in the included studies in 6, 8, and 2 papers, respectively. PEG is a synthetic material formed by polymerization of ethylene oxide, highly valuable for its hydrophilicity that facilitates exchange of cell's nutrients and waste. Despite the fact that PEG is used alone in one paper with norbornene groups (Xin et al., 2019) and combined with gelatin (Irvine et al., 2015), and alginate/GelMA (Daly et al., 2016b) in other studies, It also appears with silk fibroin (Zheng et al., 2018), poly(N -(2-hydroxypropyl) methacrylamide lactate) methacrylated (pHPMA-lactate) (Censi et al., 2011), and polycaprolactone-diacrylated (PCL-DA) (Xu C. et al., 2018). It is important to note that PEG-derived materials allow hydrogel to be photo-crosslinked, which provides better mechanical stability after bioprinting. Specifically, PEGDA presents high hydrophilicity, a bioinert structure, and lack of toxic or immunogenic responses (Zalipsky and Harris, 1997). PEGDA is used alone (Schmieg et al., 2018) and combined with alginate (Yu et al., 2018) and gelatin (Aied et al., 2018). It also appears with alginate/GelMA (Kang et al., 2017) and with alginate/GelMA/cellulose (García-Lizarribar et al., 2018). Additionally, it is combined with gellan gum (Wu D. et al., 2018), carbomer hydrogel (Chen et al., 2019), and laponite (Peak et al., 2018). Finally, PEGMA is used in two papers, one of them with alginate and a modification of PEGMA that includes a fibrinogen molecule (Maiullari et al., 2018) and the other one with alginate/GelMA/agarose (Daly et al., 2016a).

*Agarose* is used in nine papers. According to its thermal behavior, it can be compared to gelatin. In this review, it is used alone (Duarte Campos et al., 2013), combined with alginate (Blaeser et al., 2013; López-Marcial et al., 2018), collagen (Köpf et al., 2016), alginate/collagen (Yang et al., 2017), collagen/chitosan (Campos et al., 2015), and alginate/GelMA/PEGMA (Daly et al., 2016a). Additionally, it also appears combined with Matrigel (Tijore et al., 2018a) and Poly(N-isopropylacrylamide) (PNIPAAM) (Bakirci et al., 2017).

*Chitosan* is the last material included in this detailed analysis with six papers. It is a natural-obtained and biodegradable polymer very similar to other extracellular matrix components that provides great cellular viability. However, its low mechanical properties and its slow gelation make chitosan a material rarely used alone. For this reason, to solve these poor mechanical properties it is usually combined with other materials as hyaluronic acid (Kim et al., 2019), alginate/collagen (Aydogdu et al., 2019), collagen/agarose (Campos et al., 2015), and alginate/gelatin/collagen (Akkineni et al., 2016). Chitosan also appears combined with silk (Zhang J. et al., 2018) and modified with hydroxybutil groups to improve its water solubility or with oxidized chondroitin sulfate to improve its mechanical properties (Li et al., 2019).

Finally, the last three papers use polycaprolactone (PCL) (Lin et al., 2016), a blend of polyurethane (PU), PCL, poly(L-lactic acid) (PLLA) and poly[D,L-lactic acid] (PDLLA) (Hsiao and Hsu, 2018), and Thixotropic Magnesium Phosphate-based gel (TMP-BG) (Chen et al., 2018). This last hydrogel is prepared mildly (gelling method) with inorganic compound and thixotropic features that obtains promising results.

#### Hydrogel Properties

##### Concentration

Maybe, concentration is the most important parameter of hydrogels for to reasons: to assure reproducibility of the experiment, and to increase printability of the hydrogel. The importance of this parameter is clear when 89.3% of all papers define the amount of each material present in the hydrogel mixture accurately. Most papers reveal researchers are trying to develop new materials/mixtures or modifying former hydrogels to get new specific properties.

In the material section, three mains polymers stand out over the rest: alginate, gelatin and GelMA (Table 1). Alginate is the most used component in hydrogel mixtures with 122 different concentrations in 52 different papers. In general, the most used concentration range is 2–4% w/v (35 papers). Specifically, the frequency of use for 2, 3, and 4% w/v of alginate is 15, 14, and 12 papers, respectively. The rest of concentration varies between 1% w/v (10 papers) and 5% w/v (7 papers). Although standard concentrations of alginate are up to 5% w/v, Markstedt et al. (2015) and Nguyen et al. (2017) use 10, 20, 30 and 40% w/v of alginate mixed with NFC and Aljohani et al. (2018) uses 18% w/v of alginate mixed with 4% w/v of gelatin and 12% w/v of agar. In summary, the range of concentration 2–4% gives alginate its better viscosity for bioprinting as it will be seen in the next section (He et al., 2016; Wu Y. et al., 2018).

**Table 1:** Concentrations of most used materials (alginate, GelMA, and gelatin).

**Concentration (% w/v)**									
**Alginate**			**GelMA**			**Gelatin**			
**<2**	**2–4**	**>4**	**<6**	**6–10**	**>10**	**<10**	**10–15**	**>15**	**References**
	•								Blaeser et al., 2013; Bakarich et al., 2014; Narayanan et al., 2016; Freeman and Kelly, 2017; Gao et al., 2017; Li et al., 2017; Schütz et al., 2017; Yang et al., 2017; Ahlfeld et al., 2018; Habib et al., 2018; Hafeez et al., 2018; He et al., 2018; López-Marcial et al., 2018; Maiullari et al., 2018; Naghieh et al., 2018; Gonzalez-Fernandez et al., 2019; Kosik-Kozioł et al., 2019
		•							Jia et al., 2014; Markstedt et al., 2015; Nguyen et al., 2017; Aljohani et al., 2018; Datta et al., 2018; Ooi et al., 2018; Yu et al., 2018; Ji et al., 2019; Yoon et al., 2019
						•			Zhang K. et al., 2016; Aied et al., 2018; Choi et al., 2018; Li et al., 2018a,c; Shin et al., 2018; Tijore et al., 2018a,b; Yan et al., 2018
				•					Schuurman et al., 2013; Bertassoni et al., 2014; Billiet et al., 2014; Costantini et al., 2016; Levato et al., 2017
	•			•					Daly et al., 2016a,b; Kosik-Kozioł et al., 2019; Zhou et al., 2019
	•	•							Gao et al., 2015; Kundu et al., 2015; Izadifar et al., 2016; Wu Y. et al., 2018
	•					•			Akkineni et al., 2016; He et al., 2016; Berg et al., 2018
			•						Suntornnond et al., 2017; Pepelanova et al., 2018; Haring et al., 2019
•									Aydogdu et al., 2019; Jeon et al., 2019
•	•								Khalil and Sun, 2009; Raddatz et al., 2018
		•				•			Wang et al., 2016; Giuseppe et al., 2017
				•	•				Ersumo et al., 2016; Gu et al., 2018
							•		Das et al., 2015; AnilKumar et al., 2019
•		•							Polley et al., 2017
•			•						Liu et al., 2018
•			•	•					Krishnamoorthy et al., 2019
•	•		•	•					Zhang X. et al., 2018
•						•	•		Ding et al., 2018
•	•						•		Chung et al., 2013
	•	•				•			Gao et al., 2018
•	•	•					•		Bandyopadhyay et al., 2018
	•							•	Ding et al., 2017
			•	•					Mouser et al., 2016
					•				Kang et al., 2017
			•				•		García-Lizarribar et al., 2018
								•	Li et al., 2018c

*Dots shows concentration of these materials which applies in each reference*.

Gelatin is the second material with 45 concentrations in 22 papers, but with heterogeneity of values. Concentrations are distributed in a range of 1–20%, being 5% w/v the most common value (5 papers) and 10% w/v (4 papers), or 15% w/v (2 papers) other common values. It is noted that gelatin provides good thermoresponsive properties to hydrogel, but its concentration is highly dependent on the bioink application.

Finally, there are a total of 43 concentrations of GelMA in 21 papers distributed from 1% up to 20% w/v, being the most usual 10% w/v (12 papers). This concentration is followed by 5% w/v (5 papers) and 1, 6, and 20% w/v (3 papers each). Overal, the most used concentrations are 2–4, 5, and 10% w/v for alginate, gelatin, and GelMA, respectively.

##### Viscosity

This parameter can be considered an important factor for hydrogel printability. As it is known, viscosity indicates fluidity and for this reason it is very important for hydrogel extrusion. So, the more the viscous, the more the inner pressure of hydrogel during the extrusion process and increased cell damage. Pepelanova et al. (2018) proposes shear-thinning hydrogels to get an easy filament deposition during the printing process and a high shape fidelity after printing (low shear stress). However, only 12.1% of analyzed papers details viscosity or perform rheological tests of hydrogels.

He et al. (2016) performs tests with a mixture of alginate/gelatin to established a “300–30,000 cps” as the optimum range of viscosities for this kind of hydrogels. Other tests performed by Campbell et al. (2015) with a mixture of collagen/alginate recommend a viscosity higher than “2,000 cps” to maintain shape fidelity. Raddatz et al. (2018) studies some alginate concentrations and their viscosities which vary from 13.5 mPa·s (0.5% w/v) to 2,156 mPa·s (4% w/v). As said before, these viscosities are obtained with the most used concentration of alginate. Hence, according to these range of concentrations that change viscosity of hydrogel, stiffness can be modified for a proper balance between good shape fidelity (harder hydrogels) and better printability (softer hydrogels). Finally, other authors show results of hydrogel behavior in graphics, but do not provide specific values of viscosity obtained from a rheological study of a non-Newtonian fluid (Jia et al., 2014; Das et al., 2015; Pepelanova et al., 2018; Aydogdu et al., 2019; Jeon et al., 2019).

### Extrusion-Based Bioprinting

#### Bioprinting Parameters

Bioprinting parameters can be defined as those bioprinter settings (firmware inputs) needed to properly produce bioprinted structures. In this sense, only a specific range of values are adequate for bioprinting and its selection is a key factor to obtain viable bioprinted structures. However, these values are highly dependent on the hydrogel composition, so they should be carefully selected in each case. An important feature of hydrogel is printability that was no analyzed in this review because it is rarely used (He et al., 2016; Gao et al., 2018). It is defined in three levels of meaning according to viscosity (shear thinning property), curation (cross-linking), and biofabrication window (range of bioprinting parameters) (He et al., 2016). Some objective metrics to measure printability and the printability window are *Pr* (He et al., 2016), extrudability, extrusion uniformity, and structural integrity (Gao et al., 2018), but only few papers used them. For this reason, the most relevant bioprinting parameters have been selected to be analyzed in this review: cartridge temperature, bed temperature, printing pressure and printing speed.

##### Cartridge temperature

In this review, cartridge temperature is defined as the internal temperature of the cartridge/printhead and it is inversely related to hydrogel viscosity. Thus, the higher the temperature, the lower the viscosity, inducing a less shear stress decreasing cell damage. Although we are using this terminology for better understanding, *printing temperature* it is commonly used in many papers to refer the same concept. Only 45% of all the analyzed papers indicate their cartridge temperature. In this sense, up to 65 papers lack this critical parameter in its methodology. We grouped papers in five different ranges: below 20, 20–30, 30–40, above 40°C, and room temperature.

The most usual range of cartridge temperature is 30–40°C with 20 papers that use cell-laden hydrogels. In this sense, this range is focused on cells incubation temperature (37°C) together with those materials that can be used with this temperature, such as alginate, gelatin, agarose, or GelMA (13 papers) (Schuurman et al., 2013; Du et al., 2015; Fan et al., 2016; He et al., 2016; Narayanan et al., 2016; Giuseppe et al., 2017; Hafeez et al., 2018; López-Marcial et al., 2018; Pepelanova et al., 2018; Raddatz et al., 2018; Wu D. et al., 2018; Haring et al., 2019). The remaining papers use 30°C (Suntornnond et al., 2017), 35°C (Aljohani et al., 2018; Noh et al., 2019), 36°C (Blaeser et al., 2013), 38°C (Campos et al., 2015), 39°C (Blaeser et al., 2013), 40°C (Jia et al., 2014), and a range of 35–37°C (Wang et al., 2016).

On the other hand, the range of 20–30°C appears in 12 papers (Bakarich et al., 2014; Das et al., 2015; Irvine et al., 2015; Kosik-Kozioł et al., 2017; Bandyopadhyay et al., 2018; Berg et al., 2018; Cochis et al., 2018; Contessi Negrini et al., 2018; Datta et al., 2018; Gao et al., 2018; Li et al., 2018b,c; Naghieh et al., 2018). Furthermore, temperatures below 20°C are used in eight papers (Izadifar et al., 2016; Ren et al., 2016; Zhang K. et al., 2016; Celikkin et al., 2018; Gu et al., 2018; Law et al., 2018; Li et al., 2018c; Yan et al., 2018). The range above 40°C uses synthetic hydrogels with post-printing cell addition at 93°C for PCL (Stichler et al., 2017) and at 250°C for PNIPAAM polymers (Bakirci et al., 2017). However, Costantini et al. (2016) is the only author that use a *low temperature* without a specific value.

Other authors define cartridge temperature as room temperature. In our opinion, this definition can lead to misunderstandings due to the existence of different regulatory frameworks for this concept (e.g., 20–25°C for the USP-NF or 15–25°C for the European Pharmacopeia). In this review, there are 6 papers that use room temperature for their cartridge temperature (Chung et al., 2013; Kundu et al., 2015; Li et al., 2017; Zheng et al., 2018; AnilKumar et al., 2019; Ji et al., 2019). Hence, this indistinct setting of temperatures makes reproducibility of their experiments difficult, since small variations of this parameter can significantly modify the hydrogel behavior during the extrusion process as commented by Mouser et al. (2016).

Some other studies use more than one temperature or an extra wide range of temperatures that do not fit in our selected ranges. First, Mouser et al. (2016) used from 15 to 37°C in different tests to obtain the yield stress for each concentration or temperature of its GelMA/gellan gum hydrogel. Second, Daly et al. (2016a) uses 37/28/21/21°C for each component of an agarose/GelMA/alginate/PEGMA hydrogel, respectively. Third, Xu X. et al. (2018) uses from 4 to 30°C to variate viscosity of a gelatin/oxidized nanocellulose hydrogel. And finally, Mouser et al. (2017) uses 37° and 80°C for each component of a HAMA/PCL hydrogel.

##### Bed temperature

Although bed temperature can suppose a thermal shock for hydrogels and cells when high differences between bed and cartridge temperature are present, only 33 papers define this parameter in their studies. This means that most of the authors consider this information non-relevant. The temperature range is wide open from −80°C to more than 70°C. There are also five studies that defined bed temperature as room temperature (Kundu et al., 2015; Giuseppe et al., 2017; Li et al., 2017; Raddatz et al., 2018; AnilKumar et al., 2019). As said before, this indistinct temperature definition must be avoided. Specifically, Raddatz et al. (2018) sets a maximum temperature of 40°C although they use room temperature. In order to categorize this parameter, we grouped papers in five different ranges: below 0, 0–20, 20–30, 30–40, and above 40°C. According to this, there are two temperature ranges widely used: 0–20°C (9 papers) (Jia et al., 2014; Das et al., 2015; He et al., 2016; Köpf et al., 2016; Ren et al., 2016; Wang et al., 2016; Li et al., 2017, 2018c; Gu et al., 2018; Xu X. et al., 2018) and 30–40°C, being 37°C the most used temperature (5 papers) (Duarte Campos et al., 2013; Fan et al., 2016; Lin et al., 2016; Narayanan et al., 2016; Law et al., 2018), while Celikkin et al. (2018) uses 37 and 40°C, and the rest uses one temperature between this range (3 papers) (Bakirci et al., 2017; Mouser et al., 2017; Noh et al., 2019). Other bed temperature ranges are less commonly used: 20–30°C (5 papers) (Billiet et al., 2014; Ding et al., 2017, 2018; Contessi Negrini et al., 2018; Wu D. et al., 2018), below 0°C (2 papers) (Béduer et al., 2018; Choi et al., 2018), and above 40°C (2 papers) (Daly et al., 2016a,b). On the other hand, some extreme values of the whole range are used by Béduer et al. (2018) with carboxymethylcellulose hydrogel (−80°C) and Daly et al. (2016a) with a mixture of agarose/alginate/GelMA/PEGMA hydrogel (70°C).

In order to understand the importance of heating systems, Ahn et al. (2017) perform some interesting experiments that include not only a heated bed, but even an upper heated system over the nozzle. Specifically, they use a 23°C non-heated bed, a 30°C heated bed, a 32°C upper heating system, and a 36°C heated bed with upper heating system. These results are quite interesting, mainly because they enhance the importance of bed temperature, but additionally they propose a broad heated printing volume that can be controlled using a closed chamber or a specific heating system. They conclude that non-heated bed obtains the worst shape fidelity, but a combination of heated bed with upper heated system improved the shape fidelity of the bioprinted structures and got the best results in its experiments.

##### Printing pressure

Many authors (55) do not inform about printing pressure used during their experiments or use the indistinct term *low pressure* (Maiullari et al., 2018). From our point of view, this parameter is critical for a proper management of live cells during bioprinting. Additionally, it is important to note that there is no pressure unit defined for bioprinting. Although most studies use Pa, other studies use bar, psi, N/mm^2^ (Narayanan et al., 2016) or mTorr (Bakirci et al., 2017) units. In order to compare all the papers, all units were converted to SI (kPa) in this review.

Some authors use several pressures in their studies, so 127 different printing pressures were obtained. Table 2 shows these printing pressures grouped into ten different ranges: below 60, 60, 60–100, 100, 100–200, 200, 200–300, 300, 300–1,000, and above 1,000 kPa. All pressures vary between 5·10^−4^ and 4.7·10^5^ kPa, but the most used range is from 5·10^−4^ to 400 kPa (103 entries).

**Table 2:** Pressure ranges for all papers that studied this setting parameter.

**Range**	**References**
<60 kPa	Chung et al., 2013; Irvine et al., 2015; Kundu et al., 2015; Markstedt et al., 2015; He et al., 2016; Izadifar et al., 2016; Lin et al., 2016; Bakirci et al., 2017; Levato et al., 2017; Li et al., 2017, 2018a; Nguyen et al., 2017; Aljohani et al., 2018; Berg et al., 2018; Celikkin et al., 2018; Habib et al., 2018; Naghieh et al., 2018; Pepelanova et al., 2018; Raddatz et al., 2018; Wu Y. et al., 2018; Zhang J. et al., 2018; AnilKumar et al., 2019; Haring et al., 2019; Kiyotake et al., 2019
60 kPa	Bakarich et al., 2014; Daly et al., 2016a; Lin et al., 2016; Zhang K. et al., 2016; Ahn et al., 2017; Gao et al., 2018; Li et al., 2018a,c; Yan et al., 2018
60–100 kPa	Bakarich et al., 2014; Schütz et al., 2017; Gao et al., 2018; Gu et al., 2018; Li et al., 2018b,c; Raddatz et al., 2018; Zhang J. et al., 2018; Haring et al., 2019
100 kPa	Irvine et al., 2015; Kosik-Kozioł et al., 2017; Mouser et al., 2017; Schütz et al., 2017; Li et al., 2018a,c; Tijore et al., 2018b; Gonzalez-Fernandez et al., 2019; Krishnamoorthy et al., 2019; Zhou et al., 2019
100–200 kPa	Khalil and Sun, 2009; Daly et al., 2016a; Kim et al., 2016; Giuseppe et al., 2017; Levato et al., 2017; Ahlfeld et al., 2018; Gu et al., 2018; Hafeez et al., 2018; Li et al., 2018c; Wu Y. et al., 2018; Ji et al., 2019; Kiyotake et al., 2019; Noh et al., 2019
200 kPa	Das et al., 2015; Daly et al., 2016a,b; Wang et al., 2016; Ahlfeld et al., 2018; Chen et al., 2018; Law et al., 2018; Gonzalez-Fernandez et al., 2019; Zhou et al., 2019
200–300 kPa	Das et al., 2015; Ahlfeld et al., 2018; Li et al., 2018c; Schmieg et al., 2018; Yu et al., 2018; Ji et al., 2019
300 kPa	Kim et al., 2016; Wang et al., 2016; Mouser et al., 2017; Stichler et al., 2017; Suntornnond et al., 2017; Yu et al., 2018
300–1,000 kPa	Schuurman et al., 2013; Billiet et al., 2014; Kundu et al., 2015; Ersumo et al., 2016; Izadifar et al., 2016; Zhang K. et al., 2016; Li et al., 2017, 2019; Suntornnond et al., 2017; López-Marcial et al., 2018; Ji et al., 2019
>1,000 kPa	Narayanan et al., 2016; Wei et al., 2019

Almost all pressures in the range of 400–1,000 kPa are used with alginate (Li et al., 2017; López-Marcial et al., 2018), GelMA (Schuurman et al., 2013; Billiet et al., 2014; Ersumo et al., 2016; Suntornnond et al., 2017), or hyaluronic acid (Ji et al., 2019). However, the highest pressures are used in some specific materials, such as PCL/PLCL (650, 760, and 800 kPa) (Kundu et al., 2015; Izadifar et al., 2016; Zhang K. et al., 2016), or chemically-derived chitosan (600 and 700 kPa) (Li et al., 2019).

Finally, Aljohani et al. (2018) use 0.5 and 1 Pa for a gelatin/alginate/agar hydrogel. This is a very low pressure compared with the rest of papers (>5 kPa). On the other hand, Narayanan et al. (2016) use a cell-laden alginate/PLA hydrogel at 2,000 kPa, while Wei et al. (2019) use 2.2, 2.8, 4.3, and 4.7·10^5^ kPa to print an alginate hydrogel with post-printing cell addition. It is important to note that those ranges of pressure are far away from commonly used with alginate hydrogels (10–300 kPa). However, printing pressure is no longer a critical parameter of bioprinting using post-printing cell addition. In cell laden bioprinting the pressure ranges are in accordance to cellular viability, where the data obtained corroborate this affirmation. In all papers in which there are values of printing pressure and viability, 75.3% of pressure values have a viability over 80%.

##### Printing speed

Printing speed (X-Y movement) is important because it is directly related with the total bioprinting time. Aditionally, extrusion-based controls the hydrogel flow (filament width) using mainly printing speed and printing pressure. So, printing speed appears in 65 papers with 87 different velocities that vary from 0.2 to 150 mm/s. By taking a closer look, 91% of values are in the range of 1–30 mm/s where 57% of speeds are below 10 mm/s. In fact, the most used speed is 10 mm/s (13 entries) (Kim et al., 2016; Wang et al., 2016; Nguyen et al., 2017; Ahlfeld et al., 2018; Tijore et al., 2018b; Wu D. et al., 2018; Yan et al., 2018; Zhang J. et al., 2018; Ji et al., 2019; Kiyotake et al., 2019; Krishnamoorthy et al., 2019; Xin et al., 2019) followed by 5 mm/s (10 entries) (Bakarich et al., 2014; Campbell et al., 2015; Irvine et al., 2015; Markstedt et al., 2015; Narayanan et al., 2016; Giuseppe et al., 2017; Stichler et al., 2017; Habib et al., 2018; Xu X. et al., 2018). There is a printing speed that stands out because of its high value. It is used by Gao et al. (2018) with gelatin/alginate hydrogels at 150 mm/s.

#### Cross-Linking Methods

**Figure 7:**
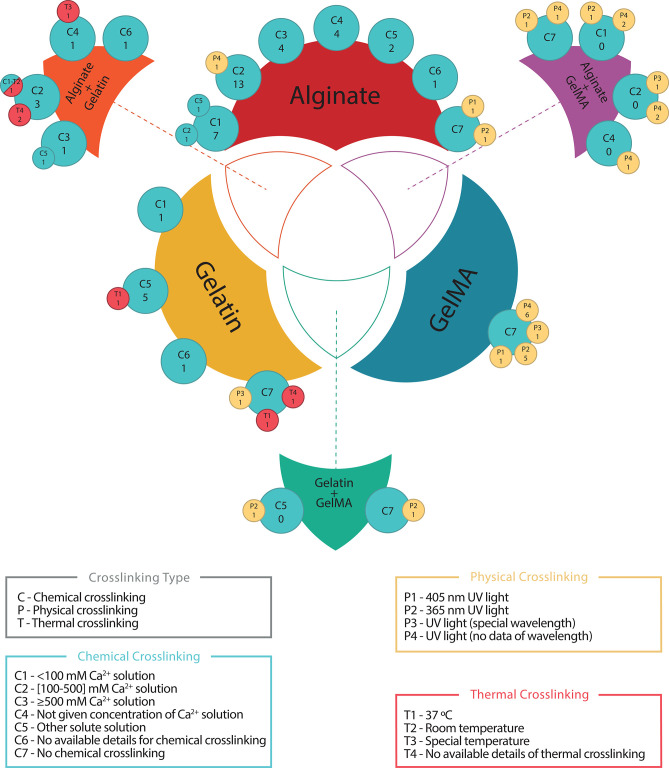
Cross-linking used for the three most common materials and its combinations. Blue, yellow, and red circles represent chemical, physical and thermal cross-linking, respectively. Superposed circles indicate that two cross-linking classifications have been combined. Each circle contains a classification codification (upper code) and the number of papers for the corresponding code combination (lower number). Chemical cross-linking has been chosen as primary classification, therefore papers not using it have been codified under C7 for graphical representation purposes and no papers are allocated to this code alone, e.g., alginate C2/13 – P4/1 (blue and yellow circles superposed) indicates that 13 papers used C2 ([100–500] mM Ca^2+^ solution) to cross-link alginate and another paper used C2 and P4 (UV light—no data of wavelength) to perform the cross-linking.

Cross-linking is usually a post-printing procedure that consists of the modification of the internal structure of the printed hydrogel to harden it and to achieve the expected mechanical properties of the bioprinted structure. It can be performed in three different ways depending on its reaction trigger: thermal (controlled by temperatures changes), chemical (controlled by the addition of reacting agents), or physical (triggered by physical procedures, usually UV light). In this sense, hydrogel composition determines the cross-linking type to use. It is a critical part in bioprinting process and surprisingly almost all authors define perfectly the materials and their protocols. Only Duarte Campos et al. (2013); Ahn et al. (2017); Contessi Negrini et al. (2018); Li et al. (2018a, 2019); Zhang J. et al. (2018) do not use any type of cross-linking. Figure 7 shows all cross-linking methods of the three most used materials: alginate, gelatin, and GelMA with its combinations. Additionally, Table 3 summarizes all analyzed studies with these three materials.

**Table 3:** The three most used materials (alginate, gelatin, GelMA, and its combinations in pairs) with its different cross-linking methods.

**Material**	**Chemical**	**Physical**	**Thermal**	**References**
Alginate	C1			Campbell et al., 2015; Markstedt et al., 2015; Narayanan et al., 2016; Freeman and Kelly, 2017; Naghieh et al., 2018; Wu Y. et al., 2018
	C1/C2			Khalil and Sun, 2009
	C1/C5			Kundu et al., 2015
	C2			Jia et al., 2014; Izadifar et al., 2016; Apelgren et al., 2017; Kosik-Kozioł et al., 2017; Nguyen et al., 2017; Polley et al., 2017; Schütz et al., 2017; Ahlfeld et al., 2018; Habib et al., 2018; He et al., 2018; Maiullari et al., 2018; Yu et al., 2018; Gonzalez-Fernandez et al., 2019
		P4		Jeon et al., 2019
	C3			Yang et al., 2017; Datta et al., 2018; Gao et al., 2018; Raddatz et al., 2018
	C4			Bakarich et al., 2014; Gao et al., 2015, 2017; Li et al., 2017
	C5			Hafeez et al., 2018; Aydogdu et al., 2019; Wei et al., 2019
	C6			Yoon et al., 2019
	C7	P1		Ji et al., 2019
		P2		Ooi et al., 2018
Gelatin	C1			Yan et al., 2018
	C5			Irvine et al., 2015; Choi et al., 2018
			T1	Tijore et al., 2018b
	C6			Shin et al., 2018
	C7	P3		AnilKumar et al., 2019
			T1	Tijore et al., 2018a
			T4	Xu X. et al., 2018
GelMA	C7	P1		Ersumo et al., 2016
		P2		Schuurman et al., 2013; Mouser et al., 2016; Levato et al., 2017; Celikkin et al., 2018; Zhou et al., 2019
		P3		Bertassoni et al., 2014
		P4		Billiet et al., 2014; Du et al., 2015; Suntornnond et al., 2017; Gu et al., 2018; Li et al., 2018cb; Pepelanova et al., 2018
Alginate Gelatin	C2			Wang et al., 2016; Ding et al., 2017, 2018
			T4	Giuseppe et al., 2017; Bandyopadhyay et al., 2018
	C2/C1		T2	Berg et al., 2018
	C3			Li et al., 2018c
	C3/C5			Akkineni et al., 2016
	C4			Chung et al., 2013
			T3	He et al., 2016
	C6			Aljohani et al., 2018
Alginate GelMA	C1	P2		Daly et al., 2016b
		P4		Daly et al., 2016a; Liu et al., 2018
	C2	P3		Zhang X. et al., 2018
		P4		Kosik-Kozioł et al., 2019; Krishnamoorthy et al., 2019
	C4	P4		Costantini et al., 2016
	C7	P2		Kang et al., 2017
		P4		García-Lizarribar et al., 2018
Gelatin GelMA	C5	P2		Haring et al., 2019
	C8	P2		McBeth et al., 2017

*Codification is at follows: C for Chemical crosslinking: C1: <100 mM Ca^2+^ solution, C2: [100–500] mM Ca^2+^ solution, C3: ≥500 mM Ca^2+^ solution, C4: Not given concentration of Ca^2+^ solution, C5: Other solute solution, C6: No available details for chemical crosslinking, and C7: No chemical crosslinking. P for Physical crosslinking: P1: 405 nm UV light, P2: 365 nm UV light, P3: UV light (special wavelength), P4: UV light (no data of wavelength). T for Thermal Crosslinking: T1: 37 °C, T2: Room temperature, T3: Special temperature, T4: no available details of thermal crosslinking*.

Thermal cross-linking is commonly used in gelatin or agarose hydrogels (16 papers). From those 16 papers, six of them perform cross-linking at 37°C (Ren et al., 2016; McBeth et al., 2017; Law et al., 2018; Tijore et al., 2018a,b; Zheng et al., 2018) and three use room temperature (Köpf et al., 2016; Lin et al., 2016; Berg et al., 2018). The rest use thermal cross-linking without specifying temperature (Fan et al., 2016; Giuseppe et al., 2017; Bandyopadhyay et al., 2018; Chen et al., 2018; Cochis et al., 2018; Xu X. et al., 2018). On the other hand, in alginate/gelatin hydrogels Berg et al. (2018) uses room temperature, He et al. (2016) uses a cool substrate, and Bandyopadhyay et al. (2018) and Giuseppe et al. (2017) use thermal cross-linking but without specific temperature. For gelatin hydrogels Blaeser et al. (2013) and Tijore et al. (2018a) use 37°C for thermal cross-linking and Xu X. et al. (2018) do not give any detail on how the thermal cross-linking performs.

Chemical cross-linking is commonly used to harden alginate, chitosan, or gelatin, but it is used with other materials too (69 papers). In general, solution with Ca^2+^ cations are used to trigger the cross-linking reaction. In this sense, 49 out of 69 papers utilize different concentrations of CaCl_2_ solution to perform the chemical cross-linking. Concentrations vary from 10 mM to 0.5 M or from 1 to 10% w/v. However, other Ca^2+^ solutes are used to perform chemical cross-linking. Specifically, Gao et al. (2018) uses CaSO_4_ or Wei et al. (2019), and Kundu et al. (2015) use NaCl_2_, also Freeman and Kelly (2017) uses CaCO_3_ and CaSO_4_. Although exposition time of the cross-linking agent is quite relevant, its definition is infrequent and, in some cases, highly different. So, Ahlfeld et al. (2018) uses 10 min while Raddatz et al. (2018) uses a 30 s mist. In order to clarify this issue for alginate Naghieh et al. (2018) performs an analysis of the cross-linking effect of CaCl_2_ at 0, 2, 4, and 24 h of exposure time. Chemical cross-linking is mostly done to alginate (52 papers) with CaCl_2_ solution (46 papers), and detailed concentrations can be seen in Figure 6. Other (non-Ca^2+^) solutions are used in two papers (Hafeez et al., 2018; Aydogdu et al., 2019) which use hydrazine and NaOH, respectively, and other two studies do not provide information (Aljohani et al., 2018; Yoon et al., 2019). Other specific cross-linking agents are genipin (Akkineni et al., 2016; Kim et al., 2016), mTgase (Tijore et al., 2018b) or 1-ethyl-3-(3- dimethylaminopropyl)-carbodiimide hydrochloride (EDC) and N-hydroxysuccinimide (NHS) (Choi et al., 2018), and MAL-PEG-MAL (Yan et al., 2018) for gelatin, or different solute for other materials like thrombin (Zhang K. et al., 2016), mushroom tyrosinase (Das et al., 2015), and oxidative reactions (Shin et al., 2018).

Finally, physical cross-linking is usually performed with the exposure of the bioprinted structures to UV light. In this sense, GelMA is the most used material with this kind of cross-linking, but all materials modified with methacrylated groups can be photo-crosslinked, such as HAMA, AlgMA or PEGMA. Among all papers (41) with this kind of cross-linking, only two of them do not use UV: AnilKumar et al. (2019) that use visible light with Rose Bengal and Riboflavin as photoinitiatorand Das et al. (2015) that use sonication procedures. In general, physical cross-linking needs a photoinitiator that triggers the reaction and some usually agents are Irgacure D-2959 or Lithium phenyl-2,4,6-trimethylbenzoylphosphinate (LAP). The most used UV wavelength is 365 nm (18 papers) followed by 405 nm (3 papers) and 312 nm (1 paper). On the other hand, Mouser et al. (2017) and Bertassoni et al. (2014) use 300–600 and 360–480 nm wavelength ranges instead of specific values, respectively. Also, Celikkin et al. (2018) use physical cross-linking without giving any kind of information. An important caution with physical cross-linking is the UV radiation effects on cells that depends mainly on wavelength and exposure time. In this sense, in 14 papers there is not information of the used wavelength. Table 3 shows that most of physical cross-linking is made to GelMA (24 papers), three papers perform physical cross-linking to alginate (Ooi et al., 2018; Jeon et al., 2019; Ji et al., 2019) and one paper use it with gelatin (AnilKumar et al., 2019). Finally, UV light power is used in a range from 2 mW to 6 W, while the exposure time varies from 10 s to 30 min.

### Post-printing Tests

#### Cellular Tests

Currently, cellular viability is one of the most common features to assess bioprinted structures that must be used on patients or drug testing. Here, post-printing analysis are focused on cellular and mechanical tests, but several biological measures appear during this review: gene expression (25 papers) that is usually related to cellular differentiation, and cell morphology (51 papers) that controls qualitatively cell shape or clustering. On the one hand, the most of gene expression studies used osteogenic- and chondrogenic-related markers such as cartilage formation genes (12 papers): ACAN (aggrecan), COL1, COL2, COL10 (collagen type I, II, X), or SOX-9. On the other hand, 37 papers conclude that there no morphological differences after bioprinting in comparison to a 2D culture and 19 papers clarify that the increasing of the stiffness, due to the increasing of the viscosity and concentration of the material or modifications of crosslinking parameters, tend cells to adopt a round shape losing its functionality (Prasad and Alizadeh, 2019). In this sense, two kinds of tests are commonly performed to determine the live/dead proportion of cells (viability tests) and its metabolic activity (metabolic tests). Table 4 compiles cellular tests grouped in these two categories, including reagents and techniques.

**Table 4:** Cellular tests carried out in the included studies.

**Test type**	**Components**		**References**
Viability tests	Calcein AM Green (live)	Ethidium homodimer (37) Orange-Red (dead)	Bertassoni et al., 2014; Das et al., 2015; Gao et al., 2015; Kundu et al., 2015; Akkineni et al., 2016; Daly et al., 2016a; Ersumo et al., 2016; Hartwell et al., 2016; Izadifar et al., 2016; Kim et al., 2016, 2019; Narayanan et al., 2016; Zhang K. et al., 2016; Ahn et al., 2017; Freeman and Kelly, 2017; Kang et al., 2017; Levato et al., 2017; Schütz et al., 2017; Stichler et al., 2017; Ahlfeld et al., 2018; Aied et al., 2018; Bandyopadhyay et al., 2018; Berg et al., 2018; Hafeez et al., 2018; Maiullari et al., 2018; Ooi et al., 2018; Shin et al., 2018; Wang et al., 2018; Wu Y. et al., 2018; Xu C. et al., 2018; Yan et al., 2018; Gonzalez-Fernandez et al., 2019; Ji et al., 2019; Kiyotake et al., 2019; Krishnamoorthy et al., 2019; Noh et al., 2019; Yoon et al., 2019
		Propidium iodide (23) Red (dead)	Chung et al., 2013; Billiet et al., 2014; Du et al., 2015; He et al., 2016, 2018; Ren et al., 2016; Wang et al., 2016; Bakirci et al., 2017; Li et al., 2017, 2018a,b; Ding et al., 2017, 2018; Gao et al., 2017; Suntornnond et al., 2017; Yang et al., 2017; Chen et al., 2018, 2019; Contessi Negrini et al., 2018; Gu et al., 2018; Pepelanova et al., 2018; Raddatz et al., 2018; Li et al., 2019
	Live/Dead assay kit* (19) Green/red (live/dead)		Censi et al., 2011; Schuurman et al., 2013; Jia et al., 2014; Markstedt et al., 2015; Costantini et al., 2016; Daly et al., 2016b; Fan et al., 2016; Nguyen et al., 2017; Aljohani et al., 2018; Habib et al., 2018; Lee et al., 2018; Li et al., 2018c; Liu et al., 2018; López-Marcial et al., 2018; Wu D. et al., 2018; Zhang X. et al., 2018; Kosik-Kozioł et al., 2019; Xin et al., 2019; Zhou et al., 2019
	Propidium Iodide** (10) Red (dead)		Blaeser et al., 2013; Duarte Campos et al., 2013; Campbell et al., 2015; Campos et al., 2015; Köpf et al., 2016; Lin et al., 2016; Giuseppe et al., 2017; Kosik-Kozioł et al., 2017; Law et al., 2018; Jeon et al., 2019
	Alamar blue (8) Blue (live)		Khalil and Sun, 2009; Irvine et al., 2015; Cochis et al., 2018; García-Lizarribar et al., 2018; Tijore et al., 2018a,b; Zheng et al., 2018; Haring et al., 2019
	Trypan blue (1) Blue (dead)		Béduer et al., 2018
	Hoechst 33342 (1) Blue (live)		AnilKumar et al., 2019
Metabolic tests	CCK8 (3) Orange (cell activity)		Hsiao and Hsu, 2018; Xu X. et al., 2018; Yu et al., 2018
	MTT (3) Purple (cell activity)		Datta et al., 2018; Zhang J. et al., 2018; Aydogdu et al., 2019
	PET activity (1)		Polley et al., 2017

(*) Unknown agents, commercial kits, (**) Different agents: Acridine orange, Fluorescein diacetate or unknown agent.

In total, 19 Live/Dead assay kits to measure the viability of bioprinted structures have been used, but none of these kits mention its composition. So, we could not perform any kind of analysis in this category. On the other hand, calcein AM has been used in 60 papers for staining alive cells and in combination with two complementary compounds: Ethidium homodimer (37 papers) or propidium iodide (23 papers) as an orange-red and red stain for dead cells, respectively (see Table 4). Additionally, propidium iodide also appears alone as viability cell marker in 10 papers, combined with fluorescein diacetate in four papers (Blaeser et al., 2013; Campbell et al., 2015; Campos et al., 2015; Jeon et al., 2019), combined with acridine orange (2 papers) (Lin et al., 2016; Kosik-Kozioł et al., 2017), and combined with other unspecified agents (4 papers) (Duarte Campos et al., 2013; Köpf et al., 2016; Giuseppe et al., 2017; Law et al., 2018). Alamar blue is a cellular viability reagent used in eight papers for staining living cells in blue color with metabolic reduction (O'Brien et al., 2000). Another test used is the trypan blue exclusion test (Béduer et al., 2018) that only stain cells with altered cell membrane, marking dead cells. It is measured as the ratio of non-stained to total cells by optical microscopy. Finally, Hoechst 33342 is a fluorescence probe that binds to the nucleus of alive cells (AnilKumar et al., 2019).

According to metabolic tests, authors measure the metabolic activity with CCK-8 (Cell Counting Kit-8) in 3 papers and MTT (3-(4,5-dimethylthiazol-2-yl)-2,5-diphenyltetrazolium bromide) in other 3 papers. CCK-8 is a colorimetric assay where intracellular dehydrogenase activity produces the soluble substrate orange formazan, while MTT is transformed in a purple insoluble salt by living cells. Also, PET (Positron Emission Tomography) measurement was made by Polley et al. (2017) with the addition of ^18^F-2-Fluor-2-deoxy-D-glucose ([^18^F]-FDG) tracer to check cell activity in volumetric geometries.

Although several studies present results of specific gene expression, cellular differentiation, or morphology, we have focused our interest in viability results. To do this, five different groups according to periods of viability measurements are established: at 0- (just after printing), 1-, 3-, 7-, and 21-days. However, data heterogeneity does not allow a statistical inference. It is important to note that comparison among studies could be relatively unfair due to many different conditions, such as: variations in bioprinted structures (grid, tubular scaffolds, discs,…), different cell lines survival characteristics (fibroblast vs. HUVECs), different measure periods (e.g., 2, 11, or 28 days), or different assay kits. Table 5 shows papers (97) that study cellular viability including material, cell type and viability. According to our analysis, the total number of papers for each group is the following: 36 papers for *0-day*, 37 papers for *1-day*, 25 papers for *3-days*, 35 papers for *7-days*, and 24 papers for *21-days*. However, some papers' time points did not fit in our five groups, and they have been grouped in the closet category as follows: 0-day and 1-day groups fit perfectly but in 3-, 7-, and 21-day are counted from 2 to 4, 5 to 11 and 12 to 28 days, respectively. In this sense, 0-, 1-, and 7-days groups are the most used by authors, although only few of them use all groups in their studies (Izadifar et al., 2016; Li et al., 2018c).

**Table 5:** Cell type with its viability grouped according to materials used in five categories (from 0 to 21 days) and expressed in percentage of cells survival.

	**Cell type**	**Cell viability (%)**					**References**	**Cell type**	**Cell viability (%)**					**References**
		**0 d**	**1 d**	**3 d**	**7 d**	**21 d**			**0 d**	**1 d**	**3 d**	**7 d**	**21 d**	
Alginate	Chondrocytes					79.00	Yang et al., 2017	MSCs		61.00			78.50	Schütz et al., 2017
		90.50					Kundu et al., 2015			85.02				Freeman and Kelly, 2017
		83.30		82.00	80.66	80.30	Kosik-Kozioł et al., 2017		71.50					Gonzalez-Fernandez et al., 2019
		72.00			85.00		Markstedt et al., 2015			88.50	86.00			Ji et al., 2019
		93.00			97.00	93.00	López-Marcial et al., 2018	HUVECs	91.00	93.00				He et al., 2018
	L929	94.00		97.00	95.00		Li et al., 2017		80.00			78.00	90.00	Maiullari et al., 2018
			97.80	95.10	91.40		Gao et al., 2017			97.00	98.00			Campbell et al., 2015
			92.90	84.70	67.10		Gao et al., 2015	ADMSC		37.50	79.00	87.50	68.50	Narayanan et al., 2016
			87.00		86.50		Ooi et al., 2018	ATDC5	85.00	82.50	82.50	82.50	87.50	Izadifar et al., 2016
		96.00	96.00	98.00	97.00		Blaeser et al., 2013	BxPC3		46.50		59.50	82.50	Habib et al., 2018
	3T3	69.00	60.00	54.21			Wu Y. et al., 2018							
	*Khalil and Sun, 2009; Jia et al., 2014; Nguyen et al., 2017; Ahlfeld et al., 2018; Hafeez et al., 2018; Raddatz et al., 2018; Jeon et al., 2019; Yoon et al., 2019													
Gelatin	HUVECs	34.60		57.60			Irvine et al., 2015	NIH3T3	95.90					Shin et al., 2018
	C2C12	93.30				69.60	Li et al., 2018ca	Bladder Ucs		93.80	78.90	81.40		Zhang K. et al., 2016
	hTMSCs		96.00		95.00	90.00	Das et al., 2015							
	*Aied et al., 2018; Tijore et al., 2018a,b; Yan et al., 2018													
GelMA	C2C12	96.00		95.00			Li et al., 2018cb	HepG2		98.61		98.72	98.92	Billiet et al., 2014
			42.00				García-Lizarribar et al., 2018			81.60	92.00	83.30		Bertassoni et al., 2014
		98.30		80.60	89.70		Zhou et al., 2019	BMSCs	91.80			92.10	94.90	Du et al., 2015
	Chondrocytes		91.80		88.50	88.30	Gu et al., 2018			75.00			90.00	Levato et al., 2017
			81.00	77.50			Schuurman et al., 2013	NIH3T3	95.00					Ersumo et al., 2016
	*Suntornnond et al., 2017; Pepelanova et al., 2018; Haring et al., 2019; Noh et al., 2019													
AlginateGelatin	MSCs	92.30					Giuseppe et al., 2017	Myoblasts	95.00		95.30			Chung et al., 2013
	L929		92.00	81.00	60.00		He et al., 2016	HDMEC		66.20			84.50	Akkineni et al., 2016
	C2C12	80.00	90.00		94.00	98.00	Bandyopadhyay et al., 2018	A549		85.00		76.25		Berg et al., 2018
	ADMSCs		88.13		90.41		Wang et al., 2016	ESCs	92.00	91.50	87.75	85.50	86.25	Li et al., 2018c
	*Ding et al., 2017, 2018; Aljohani et al., 2018													
AlginateGelMA	NIH3T3	90.00	75.00				Krishnamoorthy et al., 2019	BMSCs	88.60			85.00	76.30	Costantini et al., 2016
		89.00					Zhang X. et al., 2018		84.50			80.50	78.00	Kosik-Kozioł et al., 2019
	MSCs	81.25					Daly et al., 2016a							
	*Daly et al., 2016b; Kang et al., 2017; Liu et al., 2018													
Other	MSCs					97.97	Campos et al., 2015	MG63		50.00	80.00	78.00		Chen et al., 2018
		88.00	90.00		90.00	90.00	Xin et al., 2019			95.00	94.00	90.00		Kim et al., 2016
	Chondrocytes		93.00				Ren et al., 2016	C2C12	85.25					Contessi Negrini et al., 2018
			94.00	85.00			Censi et al., 2011	PC12		98.00	94.00			Chen et al., 2019
	HSF	90.00			85.00		Bakirci et al., 2017	NSCs	41.70					Lin et al., 2016
	NIH3T3	85.00					Wang et al., 2018	HUASMCs			90.00	82.00		Köpf et al., 2016
	ATDC5	90.00					Kim et al., 2019	BMSCs		87.00		92.00	91.00	Wu D. et al., 2018
	ADMSCs	82.00					Law et al., 2018	MC3T3		88.00		91.00	88.00	
	HTC116		88.00	83.00	52.00	40.00	Fan et al., 2016	MSCs		99.00			97	Duarte Campos et al., 2013
	BMSCs	100.00			83.00		Kiyotake et al., 2019	MG63		99.00			95.00	
	NSCs	87.00			55.00									
	*Hartwell et al., 2016; Ahn et al., 2017; Stichler et al., 2017; Cochis et al., 2018; Xu C. et al., 2018; Zheng et al., 2018; Li et al., 2019													

All studies are included in the closest period when they used a different timing. References marked with (*) make viability assay but use multiple cell lines or conditions, or not provide any value.

**Figure 8:**
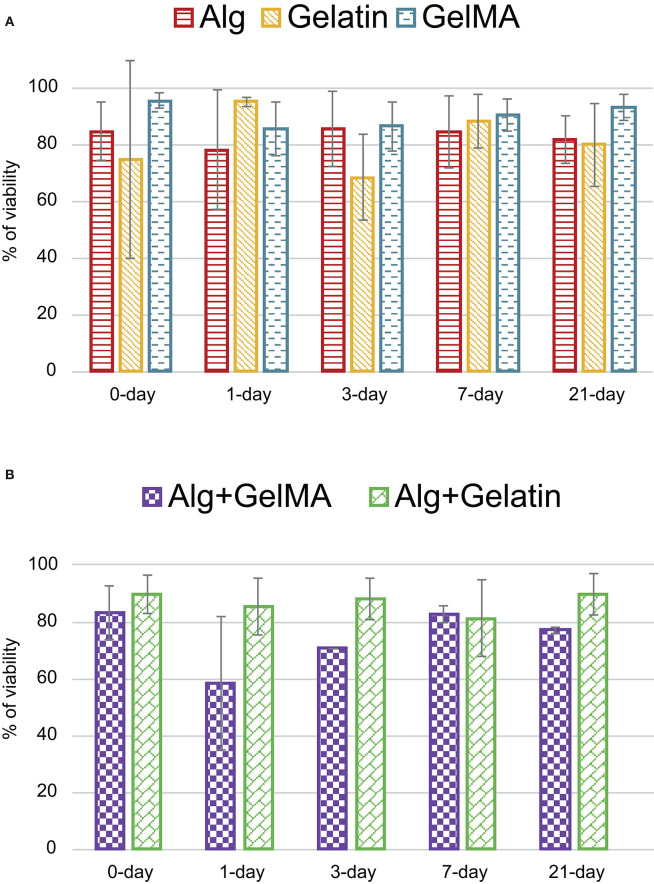
Cell viability for the most important materials: **(A)** alginate, gelatin and GelMA in the five selected periods, **(B)** material combinations for selected periods.

As mentioned before, heterogeneity of studies (different materials, cross-linking methods, temperatures, and cell lines, among others) diminishes importance of mean viability (82.70%) obtained at *0-day*. Maybe, a detailed analysis of viability by materials or cell lines could be significant, reducing the variability. For this reason, the three most used materials (alginate, gelatin, and GelMA) and two of their combinations (alginate with GelMA, and alginate with gelatin) have been selected in Figure 8 to compare their viabilities. Results show that most of the mean cellular viabilities are up to 80% with a *0-day* mean viability over 83%. This could indicate that cellular viability just after printing, has been partly sorted out. After that, *1-day* viability decreases in most cases, being more accused in the alginate-GelMA combination (58.50% from 83.05%) with the most important exception of gelatin that increases (94.90% from 74.60%). Maybe, this decreasing trend could be due to nutritional or environmental conditions of cells during this first stage. In this sense, during these first hours after the bioprinting process, cells must adapt to a new environment which in some cases, stops their growth while other provoke their death. After this stressing period, *3-days* group usually increases its cell viability, showing an adaptation to the new material in which they are embedded. Moreover, similar trend is found in *7-days* group. Finally, after *21-days* every material behaves in its own way, in GelMA and alginate/gelatin cellular viability continue its increase, while in alginate, gelatin and alginate/GelMA decrease. On the contrary, gelatin startes with a very high viability at *0-* and *1-day*, but after *3-days* decreases, reaching its minimal *21-days* later. It is noticeable that GelMA shows the best cellular viability, despite the fact that it is supposed to have the worst one even though it is synthetic (Abelardo, 2018).

Cell line depends mostly on the kind of tissue that wants to be replicated, but previous experience of authors can be a decisive too. In general, multipotent cells (e.g., Mesenchymal Stem Cells) are selected due to their differentiation potential. A total of 46 different cell lines are used in these papers. It is important to note that many papers are only qualifying cell viability using a fuzzy scale, such as good, regular or bad survival and other measures not related to viability, such as cell distribution.

**Figure 9:**
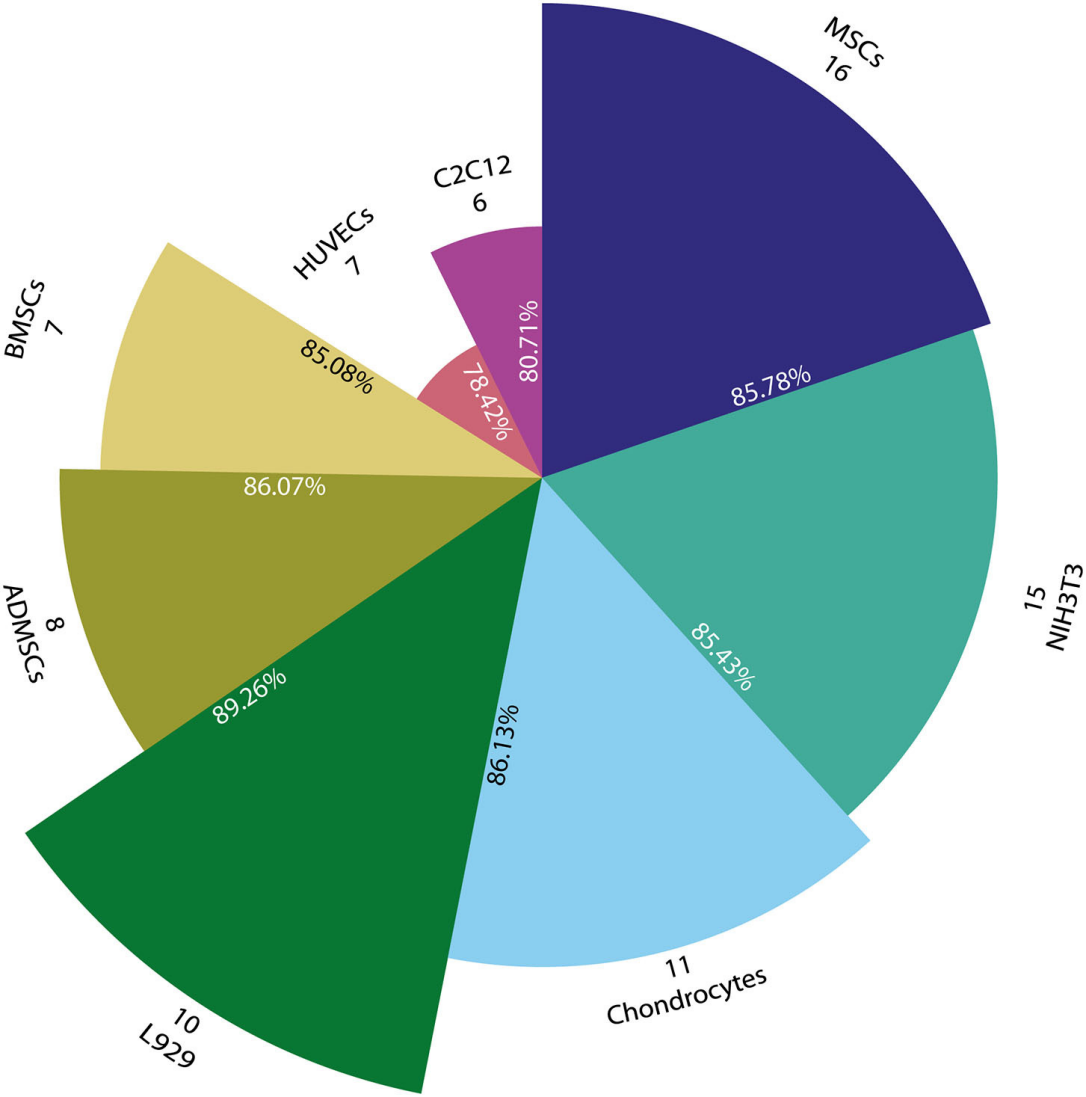
Pie chart with the most representative cell lines used for bioprinting. Angle represents number of papers that use each cell line (#) and radius indicates the mean cell viability of all related papers (%). All circle sections are proportionally scaled with viability in order to facilitate comparison.

Figure 9 shows that Mesenchymal Stem Cells (MSCs) are the most used cells (16 papers), but only some of these papers provide quantitative cell viability (9) (Duarte Campos et al., 2013; Campos et al., 2015; Daly et al., 2016a; Freeman and Kelly, 2017; Giuseppe et al., 2017; Schütz et al., 2017; Gonzalez-Fernandez et al., 2019; Ji et al., 2019; Xin et al., 2019) and the remaining reports provided a qualitative value (6) (Daly et al., 2016b; Stichler et al., 2017; Ahlfeld et al., 2018; Tijore et al., 2018b; Zheng et al., 2018; Jeon et al., 2019) with the only exception of AnilKumar et al. (2019) that does not perform any kind of test. The widely use of MSCs could be due to their ability to be differentiated into bone, cartilage, muscle, marrow, ligament and connective tissue cells (Caplan, 2011).

Additionally, NIH3T3 is used in 15 papers where some analyze percentage of cell viability (7) (Bertassoni et al., 2014; Ersumo et al., 2016; Liu et al., 2018; Shin et al., 2018; Wang et al., 2018; Zhang X. et al., 2018; Krishnamoorthy et al., 2019), others report a qualitative scale of cell viability (5) (Ahn et al., 2017; Cochis et al., 2018; Raddatz et al., 2018; Xu C. et al., 2018; Yoon et al., 2019), and some do not perform any kind of test (3). These cell types are a fibroblast cell line used mostly because of its ease of growth. After that, chondrocytes were used in 11 papers: quantitative analysis (9) (Censi et al., 2011; Markstedt et al., 2015; Ren et al., 2016; Kosik-Kozioł et al., 2017; Yang et al., 2017; Gu et al., 2018; López-Marcial et al., 2018), qualitative analysis (1) (Nguyen et al., 2017), and no test performed (1) (Mouser et al., 2016).

Other studies (10) use L929, a mouse fibroblast cell line: quantitative analysis (7) (Blaeser et al., 2013; Gao et al., 2015, 2017; He et al., 2016; Li et al., 2017; Lee et al., 2018; Ooi et al., 2018), qualitative analysis (2) (Suntornnond et al., 2017; Raddatz et al., 2018), and no test performed (1) (Polley et al., 2017). Adipose-derived mesenchymal stem cells (ADMSCs), which are a cell line used because it is easily obtained from patients (Fernández et al., 2018), appear in 8 papers: quantitative analysis (5) (Campos et al., 2015; Kim et al., 2016; Narayanan et al., 2016; Wang et al., 2016; Law et al., 2018), and qualitative analysis (3) (Jia et al., 2014; Kang et al., 2017; Pepelanova et al., 2018). Bone marrow stromal cells (BMSCs) and Human umbilical vein endothelial cells (HUVECs) are used in 7 different papers: quantitative analysis (6) (Campbell et al., 2015; Irvine et al., 2015; He et al., 2018; Liu et al., 2018; Maiullari et al., 2018; Ji et al., 2019), and qualitative analysis (1) (Suntornnond et al., 2017). On the other hand, C2C12 cells (a myoblast cell line) are used in 6 papers, all of them analyzed quantitatively (Bandyopadhyay et al., 2018; Contessi Negrini et al., 2018; García-Lizarribar et al., 2018; Li et al., 2018a,b,c; Zhou et al., 2019).

Finally, other cells are used to a lesser extent: MG-63 (5) (Blaeser et al., 2013; Duarte Campos et al., 2013; Kim et al., 2016; McBeth et al., 2017; Chen et al., 2018), MC3T3 (4) (Peak et al., 2018; Wu D. et al., 2018; Yu et al., 2018; Noh et al., 2019), NHDF (3) (Ding et al., 2017, 2018; Choi et al., 2018), 3T3 (3) (Wu Y. et al., 2018; Xu X. et al., 2018; Zheng et al., 2018), ATDC5 (3) (Izadifar et al., 2016; Hafeez et al., 2018; Kim et al., 2019), NSCs (2) (Lin et al., 2016; Kiyotake et al., 2019), Human Fibroblast (2) (Béduer et al., 2018; Zhang J. et al., 2018), PC12 (2) (Chen et al., 2019; Haring et al., 2019), HepG2 (2) (Bertassoni et al., 2014; Billiet et al., 2014), and iPSCs (2) (Nguyen et al., 2017; Maiullari et al., 2018).

Hence, the five most used cell lines are all mesenchymal cell-related. Some studies use more than one kind of cells, e.g., Liu et al. (2018) that use four different cell lines in its study. In this case, they examine cellular activity of these cell types in the GelMA cores of GelMA/alginate core/sheath microfibrous constructs. All these cell types that only appear in a specific paper are excluded from this detailed analysis. Additionally, a cell viability analysis grouped by the most used cell lines is also performed (Figure 9). In this sense, the eight most used cell lines are analyzed, but only papers with a quantitative analysis of viability are included.

In summary, all studies show a relatively high viability probably because journals perform quality controls in order to publish papers with acceptable results. In this sense, MSCs obtains a mean viability of 85.78 ± 10.2% while NIH3T3, other mesenchymal cell line, shows a similar cells survival of 85.43 ± 10.3%. In the same way, Chondrocytes (86.13 ± 6.46%), ADMSCs (86.07 ± 11.6%), and BMSCs (85.08 ± 4.95%) obtain similar viabilities. On the other hand, L-929 cells demonstrate a viability of 89.26 ± 7.42%, revealing to be the most resistant cells, in terms of viability, in all the included studies. This result is predictable, taking into consideration that L-929 cells derive from an immortalized cell line (Earle et al., 1943). Finally, HUVECs and C2C12 show less viability than other cell lines with 78.42 ± 19.3 and 80.71 ± 19.6%, respectively. However, this last analysis presents a high standard deviation, probably due to the small sample. A similar analysis could be provided linking materials and type of cells with its viability. But not enough number of combinations of different materials with cell types can assure a cause-effect relation.

#### Mechanical Tests

This section compiles different tests that obtain mechanical properties of bioprinted structures. In all 66 out of 118 papers perform different mechanical tests, but we have focused our interest into the five most used: (1) compressive stress, (2) Young or elastic modulus, (3) compression modulus, (4) yield stress, and (5) ultimate tensile strength (UTS). To obtain these mechanical properties, tests are done to multiple different materials, where alginate (51% of papers), GelMA (27%), and gelatin (15%) are the most common.

Compressive stress is defined as the stress on materials that leads to a smaller volume (14 papers). Kim et al. (2016) is the only study where graphs are used to show results. We group papers into five different ranges: below 10, 10–40, 40–100, 100–400, and 1,200–4,100 kPa. With the most usual values in the 10–40 kPa subrange (9 papers), values lower than 10 kPa are the second most usual subrange (5 papers), and the rest of data is distributed in 40–100, 100–400 kPa, and 1,200–4,100 subranges (3, 2, and 3 papers, respectively).

Three authors study compressive stress of different materials. On the one hand, Daly et al. (2016a) obtain 4, 16, 20, and 36 kPa for alginate, agarose, PEGMA, and GelMA hydrogels, respectively. On the other hand, Aydogdu et al. (2019) perform their tests in the 1,200–1,400 kPa range. They use different mixtures of alginate, collagen, chitosan, Hlh (Halomonas levan), PLA, and b-TCP to create different hydrogels, obtaining 1,240, 1,290, 1,380, 1,490, 1,540, and 1,590 kPa for each hydrogel. Additionally, Ahlfeld et al. (2018) obtain 16.9, 1,300, and 4,100 kPa for an alginate, methylcellulose, and CMC hydrogel, respectively.

Young modulus is defined as the mechanical property to measure the stiffness of a solid material and it is the most analyzed parameter. Hence, the Young modulus defines the relationship between stress (force per unit area) and strain (proportional deformation) in a material in the linear elasticity regime of a uniaxial deformation. Approximately half of papers (35) study this property and some of them comment the expected result for a specific tissue, e.g., 4 MPa for cartilage tissue (Censi et al., 2011). In this case, six authors show their results in graphs and the quantitative results are within 0–100, 100–400, and above 400 kPa ranges. In general, the whole range varies from 0.15 to 8.3·10^5^ kPa, most of the values are in the range 0–100 kPa with 23 papers, followed by 100–400 kPa (10 papers), and above 400 kPa (3 papers). Alginate is the most studied material (16 papers). Several kinds of are present: Wei et al. (2019) analyzes complete mechanical properties of cross-linked wet and dry alginate scaffolds, other authors study cross-linked hydrogels in different times (Khalil and Sun, 2009; Naghieh et al., 2018; Shin et al., 2018), or in different concentrations (Bertassoni et al., 2014; Mouser et al., 2016; Naghieh et al., 2018; Krishnamoorthy et al., 2019). It is important to note the work of Aljohani et al. (2018) that analyzes alginate blended with gelatin and agar, obtaining the highest values of the Young modulus with 5.7·10^5^, 6.1·10^5^, 6.4·10^5^, and 8.3·10^5^ kPa for an alginate, gelatin, agar, and alginate/gelatin/agar hydrogel, respectively.

Compression modulus is the ratio of mechanical stress to strain in an elastic material when that material is being compressed, i.e., the compressive force per unit area divided by the change in volume per unit volume. In this case, 17 papers perform 57 tests, where four papers performed this test giving their results only in graphs. The most usual range is 10–100 kPa with 11 papers and 32 different compression values. However, two studies obtain higher values than 1 MPa: Daly et al. (2016b) with 1,402 kPa using an alginate/PCL blend and Ahlfeld et al. (2018) with 36.7, 3.07·10^4^, and 5.2·10^4^ kPa for an alginate, CPC, and methylcellulose hydrogels, respectively. Additionally, some studies analyze the compression modulus with different concentrations (Chung et al., 2013; Schuurman et al., 2013; Giuseppe et al., 2017), several cross-linking agents for alginate, PEGDA, and GelMA (Kang et al., 2017), and different cell lines (Levato et al., 2017).

Ultimate tensile strength (UTS) is the maximum stress that a material can withstand while being stretched or pulled before breaking. This property appears in 12 papers and nine of them use alginate in their hydrogel. In this case, when alginate is used (mixed or not with another component) UTS values are within 40–500 kPa range with concentrations of 2–5%w/v.

Finally, yield stress is little studied (3 papers) and most of them provide graphical results. Bandyopadhyay et al. (2018) obtain a yield stress of 3,350 kPa using an alginate/gelatin/collagen hydrogel. Lastly, Li et al. (2018b) study the ultimate shear stress (12 kPa) in a GelMA/Carrageenan hydrogel.

As exposed before, all these properties are highly dependent of pre- and printing process. In this sense, concentration, and crosslinking parameters, are the parameters that affect the most to mechanical stability of bioprinted structure. This is evident when the main modifications in studies relating mechanical properties are made with changes in these parameters. For example, changes in concentration to analyze how it affects the mechanical properties are made in 11 papers, 4 of them young modulus is observed (Bertassoni et al., 2014; Mouser et al., 2016; Naghieh et al., 2018; Krishnamoorthy et al., 2019), in 4 papers UTS (Gao et al., 2015; Yang et al., 2017; Bandyopadhyay et al., 2018; He et al., 2018), and in 3 compressive modulus (Chung et al., 2013; Schuurman et al., 2013; Giuseppe et al., 2017). All of them obtain the same conclusion: mechanical stability rises when the concentration increases. Mechanical properties are also influenced by crosslinking parameters. In this sense Giuseppe et al. (2017) proposed a 15 min time of exposition of Ca^2+^ for alginate/gelatin blend after measuring different time point and analyze it compressive modulus, noting that with higher time of exposition modulus increases. Also, Kang et al. (2017) made modifications in their photoinitiator, its concentration and power of UV irradiated. In the same way as before, the higher the concentration and the irradiation power the higher the stiffness.

## Conclusions

This article is a systematic review of hydrogel implications during bioprinting process, including a descriptive statistical analysis of materials, bioprinting parameters, mechanical tests, and viability assays.

Maybe, the omission of relevant bioprinting parameters is one of the most important drawbacks detected in most of the papers, making the reproducibility of their results difficult. Obviously, many research fields are involved in bioprinting, so it is possible that authors focused their interest on those parameters directly related to its scope, playing down the rest of essential information. For this reason, we propose some suggestions to solve this problem in section “Recommendations and future works.”

First, alginate is the most commonly used material followed by gelatin and GelMA. For this reason, the concentration and cross-linking analysis are highly influenced by these three materials. Here, we show that the most used concentrations are 2–4, 5, and 10% w/v for alginate, gelatin, and GelMA, respectively. Likewise, most cross-linking methods for alginate are chemical and based on Ca^2+^ cations, while 37°C is the most common temperature for thermal cross-linking of gelatin, and UV light is the standard physical cross-linking of GelMA.

Secondly, cell-laden hydrogels are the most used. Consequently, cartridge temperature is usually defined in the range of 30–40°C (allowing cell survival) and the printing pressure at 100–200 kPa (reducing cell stress). Obviously, the addition of cells after hydrogel bioprinting minimize the importance of these parameters.

Finally, MSCs are the cell line most used in combination with hydrogels. In general, good viability results are obtained with all cell lines. Regarding mechanical tests, the Young modulus is widely used in bioprinting, although there is no consensus on the most important mechanical property of each bioprinted structure.

## Recommendations and Future Works

In our opinion, those missed bioprinting parameters are usually related to a poor reproducibility. Moreover, inappropriate evaluation tests may cause an unfair comparison of results. For this reason, some guidelines and recommendations are detailed below. Additionally, in order to facilitate reading and understanding for future papers, the International Systems of Units (SI) must be used.

Hence, we strongly recommend defining the following parameters in all studies. *Concentration* of materials and protocols to prepare hydrogels should be fully detailed and could be complemented with its viscosity. Although *cartridge temperature* and *printing pressure* are two essential parameters needed to set the bioprinter, *bed temperature* and *printing speed* will increase the reproducibility of the study. A quantitative measure of printability or the hydrogel printability window will facilitate its practical use. Additionally, in the cross-linking step, *concentration* for chemical-based cross-linking, *temperature* for thermal-based cross-linking, *light wavelength*/*power* for physical-based cross-linking must be defined. Furthermore, the *exposition time* must be defined for all three cross-linking types. On the other hand, cellular tests must include the identification of *cell line* and *assay-kit* information with quantifications at different time points (*0, 1, 3, 7, and 21-days*). And finally, those studies whose bioprinted structures have a specific clinical application must perform mechanical tests to mimic the tissue/organ properties (e.g., compressive stress for cartilage tissue).

In summary, due to time and space restrictions, this review could not analyze all the information available in the selected papers. Thus, future works could focus on comparing results of commercial vs. homemade bioprinters (cell viability, mechanical behavior), analyzing other rheological properties (swelling ratio, surface tension), printability vs. precision, or degradation speed for different hydrogels.

## Author Contributions

EM performed literature search, screening, data extraction, preparation of graphics, tables, and manuscript. JG-B contributed to screening, data extraction, preparation of graphics, tables, and manuscript. JP contributed to screening, data extraction, and preparation of manuscript. EL, JC, AM-G, MD, JC-A, and DT contributed to screening, data extraction, and revision work to the manuscript. FS-M performed revision work to the manuscript. All authors contributed to the article and approved the submitted version.

## Conflict of Interest

The authors declare that the research was conducted in the absence of any commercial or financial relationships that could be construed as a potential conflict of interest.
